# Case of Calculus Vesicæ; Dilatation of the Urethra; Lithotrity

**Published:** 1846-03

**Authors:** Henry Crawford

**Affiliations:** Surgeon to the Infirmary, Canterbury


*Case of Calculus Vesicæ; Dilatation of the Urethra; Lithotrity.* By Henry Crawford, Esq., Surgeon to the Infirmary, Canterbury. —The subjoined case, in which the operation of lithotrity proved unsuccessful, presents many points of surgical interest, on which account I present it to the notice of your readers.

Sarah Stone, aged 46, mother of thirteen children, was admitted into the Canterbury Hospital, October 24th, 1845. Her countenance was expressive of intense suffering; she complained of very painful and very difficult micturition, and distinctly described the sensation of a large heavy substance within her, which arrested the flow of urine, by falling against the orifice of the bladder, so that her nights were often passed in painful and fruitless efforts to relieve that organ. All the pelvic viscera suffered distressing sensations of weight and dragging, frequently aggravated into throes resembling those of parturition. The urine deposited an abundant mucous sediment, and was of an highly alkaline smell. Upon passing a sound into the bladder, a large calculus was immediately felt. She dated the commencement of her sufferings *ten years* ago, but during the last five they have become much more intense. She was ordered:—

℞. Infus. Pareiræ, oz. xij,; Acid. Nitric. dilut., dr. ij; Spir. Ætheris Nit., dr. iij. M. Capiat unciam sextis horis.

℞. Solutionis Morphiæ Muriat., Tinct. Hyoscyami, utrq. m. xx.; Mist. Camphoræ, oz. iss. M. Fiat haustus hora somni sumendus.

After a few days I gradually dilated the urethra, by means of Weiss's dilator, until I could pass my finger and feel the stone, which was readily grasped by a pair of lithotomy forceps, but the distance to which the handles of the instrument were separated, made it evident that something further was necessary to render extraction possible. I felt inclined to adopt the operation advised by Mr. Liston, under such circumstances, viz., to notch the fibres of the sphincter vesicæ on each side, afterwards employing the dilator with the view of obtaining room to extract the foreign body entire. However, after consultation with my colleagues, it was agreed to avoid the use of cutting instruments altogether, and to reduce the size of the stone by lithotrity. Accordingly, on Friday, October 31st, I proceeded to inject the bladder, which could be only imperfectly effected, and with considerable pain, on account of the irritable condition of that organ. Upon withdrawing the catheter I introduced Weiss's screw-lithotrite, with which the stone was easily seized and crushed, its diameter being, according to the index on the blade of the instrument, about two inches and a quarter; several large fragments were at the same time comminuted with but little pain to the patient. During several following days the fragments passed off freely; she expressed herself much relieved of the obstruction to micturition; the urine was longer retained, of a more healthy appearance, with less of mucous deposit or alkaline smell.

Encouraged by these favourable appearances, and aware that much remained to be done before the bladder could be cleared, I renewed my operations on the succeeding Monday, Tuesday, and Wednesday, crushing many fragments, and removing a portion of the detritus by the use of the screw-scoop. The pain chiefly complained of on each of these occasions was that arising from the injection of the bladder. I desired all that passed should be preserved, but this was not done, as much of the detritus mingled with the urinary deposit in the form of impalpable powder, and she stated that large pieces escaped from the urethra during the act of defæcation. Some large fragments which were collected shewed the composition of the calculus to be triple phosphate, with here and there a layer of the lithates.

On Friday she complained that the passage of urine was again obstructed, and a large fragment was felt by the catheter, presenting at the internal orifice of the urethra. I endeavoured to push this back into the cavity of the bladder, in order to seize it with the lithotrope, but all my efforts were ineffectual. I then thought it would be possible, after moderately dilating the urethra, to remove this fragment with the lithotomy forceps, as I had attempted to do with the entire calculus in the first instance; this I put in practice on Saturday morning, and persevered for several minutes in gentle but firm attempts at extraction ; the proceeding caused considerable pain, and I was at length reluctantly compelled to let go my hold.

On the Monday following she was very sick, which she attributed to the fright caused by an accident which had been brought into the ward on the previous day ; vomiting of all ingesta succeeded, with pain in the epigastrium, which quickly extended over the whole abdomen, and she sank with all the symptoms of peritonitis, on Saturday, November 15.

*Autopsy.*—The peritoneal surface of the stomach and intestines was coated with recently effused lymph ; the walls of the bladder were much thickened ; its cavity contracted ; its mucous membrane of a dark venous-red colour, and it contained *one* flattened irregular shaped portion of stone, weighing three drachms, but *no other fragments, or detritus.* The mucous membrane was not ulcerated, nor did it appear to be injured by the instruments employed.

*Remarks.*—The application of lithotrity to large stones has been strongly deprecated by some of our most able surgeons ; but their observations have reference chiefly to the operations on the male ; the facility afforded to the passage of fragments of considerable size through the short and dilatable female urethra, diminish the force of many of the above objections when applied to it. Had Mr. Liston's operation been adopted in the case of Sarah Stone, it may be doubted whether fatal consequences might not have followed the immense degree of dilatation required, not to mention the dreadful (though minor) evil of a permanent incontinence of urine. The unfavourable symptoms in the present case followed the second employment of the dilator, which occasioned more suffering than the lithotrite, though not carried so far as to produce even temporary incontinence of urine. The small amount of pain and temporary relief produced by crushing of the stone, together with the remarkable manner in which the bladder contrived to rid itself of so large a portion of fragments and detirtus in the above case, appear to indicate that nature is on our side in the employment of crushing, as a means of relieving the sufferer from vesical calculus ; though I admit that the question remains open whether the safer, and therefore the preferable operation to have recourse to in another similar case, would not be the old one of lithotomy, through the vagina.—***Prov. Med. and Surg. Journ.***

